# The seroprevalence of hepatitis B, hepatitis C, and human immunodeficiency virus in patients undergoing septoplasty^☆^

**DOI:** 10.1016/j.bjorl.2016.10.008

**Published:** 2016-11-17

**Authors:** Ozlem Onerci Celebi, Ela Araz Server, Bahtiyar Hamit, Özgür Yiğit

**Affiliations:** Istanbul Education and Research Hospital, Department of Otolaryngology, Istanbul, Turkey

**Keywords:** Septoplasty, HBV, HCV, HIV, Seroprevalance, Septoplastia, HBV, HCV, HIV, Soroprevalência

## Abstract

**Introduction:**

Worldwide, hepatitis B virus, hepatitis C virus, and human immunodeficiency virus are major health problems. Healthcare workers are at risk of transmitting blood–borne viruses, and surgeons have a higher risk of exposure to blood and higher rates of percutaneous injury than other healthcare workers. Septoplasty is among the 3 most commonly performed otolaryngological surgeries worldwide.

**Objective:**

To determine the seroprevalence of Hepatitis B surface antigen, Hepatitis C virus antibody, and Human Immunodeficiency Virus antibody in patients undergoing septoplasty with and without turbinate surgery under general anesthesia, and to determine if preoperative testing should be performed in such patients.

**Methods:**

This retrospective cross-sectional study included 3731 patients that underwent septoplasty with and without turbinate surgery between January 2005 and July 2015. HBsAg, anti-HCV, and anti-HIV seropositivity in the patients was evaluated retrospectively.

**Results:**

Mean age of the patients was 36 years (range: 11–81 years). In all, 117 (3.6%) patients were positive for HBsAg, 12 (0.3%) were positive for anti-HCV, and 7 (0.2%) were positive for anti-HIV.

**Conclusions:**

Education of healthcare workers combined with routine preoperative serological testing in patients undergoing septoplasty under general and local anesthesia are needed to increase awareness of hepatitis B and C, and HIV infection among healthcare workers and patients in order to decrease the transmission rate.

## Resumo

**Introdução:**

No mundo todo, o vírus da hepatite B (VHB), o vírus da hepatite C (VHC) e o vírus da imunodeficiência humana (HIV) são problemas de saúde importantes. Os profissionais de saúde correm o risco de transmitir vírus transmitidos pelo sangue, e os cirurgiões têm um maior risco de exposição ao sangue e taxas mais elevadas de lesões percutâneas do que os outros profissionais de saúde. A septoplastia está entre as três cirurgias otorrinolaringológicas mais comumente realizadas em todo o mundo.

**Objetivo:**

Determinar a soroprevalência de anticorpos HBsAg, anti-HCV e anti-HIV em pacientes submetidos a septoplastia com e sem cirurgia de concha nasal sob anestesia geral e determinar se deve ser realizado teste pré-operatório nesses pacientes.

**Método:**

Este estudo transversal retrospectivo incluiu 3731 pacientes que foram submetidos à septoplastia com e sem cirurgia de concha nasal entre janeiro de 2005 e julho de 2015. A soropositividade para HBsAg, anti-HCV e anti-HIV nos pacientes foi avaliada retrospectivamente.

**Resultados:**

A idade média dos pacientes foi de 36 anos (intervalo: 11-81 anos). Ao todo, 117 (3,6%) pacientes foram positivos para HBsAg, 12 (0,3%) foram positivos para anti-HCV e 7 (0,2%) positivos para anti-HIV.

**Conclusões:**

A educação de profissionais de saúde combinada com testes sorológicos rotineiros pré-operatórios em pacientes submetidos a septoplastia sob anestesia geral e local são necessários para aumentar a conscientização sobre a hepatite B e C e a infecção pelo HIV entre profissionais de saúde e pacientes para diminuir a taxa de transmissão.

## Introduction

Worldwide, hepatitis B virus (HBV), hepatitis C virus (HCV), and human immunodeficiency virus (HIV) are major health problems. Among the 2 billion people infected with HBV worldwide, more than 350 million suffer from chronic HBV infection.^1^, ^2^ The prevalence of HCV infection is reported to be 2.3%–3% (130–170 million people), of which 80% develop chronic infection.^3^, ^4^ Additionally, WHO reported that 2 million people were infected with HIV and that there were 1.2 million deaths related to AIDS in 2014.^5^

Healthcare workers (HCWs) are at risk of transmitting blood–borne viruses, and surgeons have a higher risk of exposure to blood and higher rates of percutaneous injury than other healthcare workers.^6^, ^7^, ^8^ It is estimated that 16,000 HCV; 66,000 HBV and 1000 HIV infections have occurred worldwide in healthcare workers in 2000 as a result of occupational exposure to percutaneous injury.^8^ Transmission of blood–borne pathogens can occur via percutaneous and mucocutaneous routes, and sometimes via exposure to other body fluids.^8^, ^9^

Septoplasty is among the 3 most commonly performed otolaryngological surgeries worldwide.^10^, ^11^ It can be performed under general or local anesthesia.^12^, ^13^, ^14^ The nose – and in particular the septum – is a vascular organ and surgeons commonly encounter bleeding at some point during septoplasty. Moreover, surgeons generally use sutures to stabilize the septum, to stabilize the perichondrium (using transfixion sutures) so that nasal packing is unnecessary, to prevent such complications as hematoma and bleeding, and to close any septal mucosa tears.^15^, ^16^ Turbinates are also vascular organs and turbinate surgery performed with septal surgery further increases bleeding.^17^, ^18^ These factors are associated with surgeon exposure to patient blood and/or secretions, which consequently increases the risk of blood borne virus transmission, especially HBV, HCV, and HIV.^19^, ^20^

At our clinic preoperative serological testing for HBV, HCV and HIV is performed in every adult patient in which elective surgery under general anesthesia is indicated, but not in patients undergoing elective surgery under local anesthesia, including septoplasty. The aim of the present study was to determine the seroprevalence of HBsAg, anti-HCV, and anti-HIV in patients undergoing septoplasty with and without turbinate surgery, and to determine if preoperative testing for HBV, HCV, and HIV should also be performed in patients undergoing septoplasty with and without turbinate surgery under local anesthesia as an additional precaution to avoid exposure to patient blood and secretions during surgery.

## Methods

This study included 3731 patients that underwent septoplasty with and without turbinate surgery between January 2005 and July 2015. HBsAg, anti-HCV, and anti-HIV seropositivity in patients admitted to the Otolaryngology Clinic for septoplasty with and without turbinate surgery was retrospectively analyzed. Preoperative blood samples were analyzed by our hospital's microbiology department. Patient age, gender, type of surgery, and serological data and hematological counts were recorded. The patients who had additional sinonasal symptoms and whose CT scan revealed sinonasal disease had endoscopic sinus surgery in addition to septoplasty and thus were excluded from the study.

The study protocol was approved by the local Ethics Committee (18/12/2015-742), and was conducted in accordance with the Declaration of Helsinki. Data frequency and mean ± SD were analyzed.

### Statistical analysis

Statistical analysis was performed using SPSS v.16.0 for Windows (SPSS, Inc., Chicago IL). Descriptive statistics are given with each value's Standard Deviation (SD). The normality of the distribution of data was determined using the Kolmogorov–Smirnov test. None of the data were normally distributed. Gender differences in age, and HBsAg, anti-HCV, and anti-HIV seropositivity were analyzed using the Mann–Whitney *U* test. The level of statistical significance was set at *p* < 0.05.

## Results

In total, 3731 patients underwent septoplasty in the otolaryngology department between 1 January 2005 and 31 December 2015. Among the patients, 3241 underwent surgery with general anesthesia, versus 490 with local anesthesia. Preoperative serological data were available only for patients that received general anesthesia; thusly, all analyses were performed with these 3241 patients’ data. Mean age of the patients was 36 years (range: 11–81 years). In total, 117 (3.6%) patients were positive for HBsAg, 12 (0.3%) were positive for anti-HCV, and 7 (0.2%) were positive for anti-HIV. All 7 patients that were anti-HIV positive were referred for further confirmation. Among the patients, 11 were aged < 18 years, all of which were negative for HBsAg, anti-HCV, anti-HIV.

Among the patients, 941 were female and 2300 were male. In all, 2% of the female patients (23/941) were positive for HBsAg, versus 4% of the males (94/2300). The HBsAg positivity rate was significantly higher in males (Mann–Whitney *U* test, *p* = 0.023). In total, 0.31% of the female patients (3/941) and 0.39% of the male patients (9/2300) were positive for anti-HCV. All 7 patients that were positive for anti-HIV were male. The anti-HIV positivity rate was 0.3% considering only the male patients. The anti-HCV and anti-HIV positivity rates did not differ significantly between genders. Serological findings are summarized in the Table 1.

**Table 1:** Serological data for HBsAg, anti-HCV, and anti-HIV.

	Total		Females (*n* = 941)		Males (*n* = 2300)		*p*
	*n*	%	*n*	%	*n*	%	
HBsAg	117	3.6	23	2	94	4	**0.023**^a^
Anti-HCV	12	0.3	3	0.31	9	0.39	0.758
Anti-HIV	7	0.2	0	0	7	0.3	0.09

a *p* < 0.05 resembles statistical significance.

No alteration or correlation was found between the hematological counting (Complete Blood Count) and the positive patients.

## Discussion

HBV, HCV, and HIV are important healthcare problems worldwide. Surgeons have a higher risk of exposure to blood and higher rates of percutaneous injury than the other healthcare workers.^6^, ^7^, ^8^ and the significant rates of morbidity and mortality due to these viruses suggest surgeons should use all precautions possible to avoid exposure to patient blood and secretions during surgery. Percutaneous injury occurs during many surgical procedures, varying in frequency with surgical specialty, and the 75% of blood–borne pathogen exposure occurs during surgery.^20^, ^21^ It was reported that 5.5% of percutaneous injuries occur in otolaryngological practice.^21^ The suture needle was reported to be the instrument most associated with percutaneous injury and exposure to blood–borne pathogens, accounting for 50% of all surgical injuries, followed by sharp instruments (34%) – primarily the scalpel blade (6.9%); the suture needle and scalpel blade are the 2 most commonly used surgical instruments during septoplasty.^20^, ^21^ Incision, suturing, wound closure, and increased bleeding are all associated with surgeon exposure to blood and secretions.^20^, ^21^

It was reported that vaccination against HBV, use of protective glasses and gloves, double gloving, and blunt suture needles are the precautions necessary to protect against blood and body fluid exposure.^7^, ^21^, ^22^, ^23^, ^24^ Whether or not preoperative testing of patients for HBV, HCV, and HIV can further decrease the risk of transmission remains unclear; in countries with low infection rates, preoperative testing is not common because it is not thought to be cost-effective, whereas in countries with high infection rates it is strongly recommend that preoperative testing be performed.^23^, ^25^ Transmission of infections via blood occurs at a higher rate in developing countries than in developed countries, and occupational blood exposure is of great concern in developing countries.^19^ Turkey is a developing country in which the seropositivity rates were reported to be 0.52%–4.19% for HBsAg, 0.1%–1% for anti-HCV, and 0%–0.1% for anti-HIV, and the present seroprevalence findings of the current study are similar to those reported earlier for Turkey.^26^, ^27^, ^28^ Turkey is an intermediate-endemic region for HBV and a low-endemic region for HCV.^4^, ^29^, ^30^, ^31^, ^32^ Moreover, HIV/AIDS is currently considered to be an emerging disease in Turkey.^33^ As such, at our surgical department we request preoperative serological testing including HBsAg, anti-HCV, and anti-HIV, in all patients undergoing surgery under general anesthesia.

The preoperative prevalence of HBV in the present study's septoplasty patients was significantly higher in males (4%) than in females (2%), as previously reported, which we think could be due greater social mobility and freedom among males in developing countries, especially in rural areas, making the male population more prone to contracting the infection^34^, ^35^; however, there are other studies from developing countries showing higher rates of infection in females.^36^

Our department frequently performs septoplasty; 3731 patients underwent the procedure with and without turbinate surgery between 2005 and 2015, of which 3241 received general anesthesia and 490 received local anesthesia. Local anesthesia is a safe and cost-effective technique for sinonasal surgery in selected patients when pain can be prevented and/or treated during surgery.^12^, ^37^, ^38^ Our department generally uses general anesthesia during septoplasty (3241 of 3731 patients) and all patients undergoing septoplasty under general anesthesia undergo preoperative serological testing, including HBsAg, anti-HCV, and anti-HIV; however, serological testing is not performed in our patients undergoing septoplasty under local anesthesia. Local anesthesia is associated with less bleeding during septoplasty than general anesthesia^37^, ^38^; however, bleeding can still be a problem for some patients under local anesthesia. Our patients that receive local anesthesia generally have a high risk for general anesthesia, including hypertensive patients and patients that use blood-thinners for cardiac disorders. These patients have a tendency for bleeding even under local anesthesia, putting the surgeon at risk of exposure to blood. Additionally, although local anesthetic agents are effective pain relievers, it is reported that not all patients are satisfied with the pain medication they get from local anesthesia.^12^ Patients that are sensitive to pain tend to make sharp movements in response to pain during surgery, jeopardizing positioning of the scalpel blade and sutures.^14^, ^39^ Moreover experiencing pain increases blood pressure, making patients more prone to bleeding. Turbinate surgery also increases the risk of bleeding. All of these factors also are significant risk factors for surgeon exposure to blood and percutaneous injury during septoplasty under local anesthesia, which suggests that preoperative serological testing in patients receiving local anesthesia could be routine.

We obtain the CT scan if the patient reports any symptoms related to sinus disorders. Of the patients who we obtained CT scan, the patients who had sinus disease were excluded from the study and had endoscopic sinus surgery performed in addition to septoplasty. We wanted to exclude these because we only wanted to include the most widely performed procedure (septoplasty) and make the surgeons aware that although it is commonly performed and a relatively easy and straightforward surgery, it should be kept in mind that even the most common and basic surgical procedure puts the surgeon under risk of transmitting blood–borne pathogens. When thinking about the complications of septoplasty or any surgery, transmission of the infection should always be kept in mind.

As mentioned above, preoperative serological testing, including HBsAg, anti-HCV, and anti-HIV, is performed in all of our patients undergoing surgery under general anesthesia. In a developing country with a high prevalence of these viruses, such testing alerts surgeons, nurses, and the entire surgical team to the need for extra caution during surgery. In addition, preoperative serological testing facilitates informing our patients that are unaware of their seropositivity and early detection of infection in patients in which the associated diseases would otherwise go undetected, which helps limit the spread of virus in the community. The present study's seropositive patients were referred for further investigation and were encouraged to take all necessary precautions to prevent transmission to other individuals. These precautions are especially important, as worldwide a high proportion of occupationally acquired HCV, HBV, and HIV infections occur in developing countries such as Turkey, in which healthcare workers are exposed to a patient population with a higher prevalence of blood–borne viruses than in developed countries.^19^

None of the 7 patients who were positive for anti-HIV were aware of their disease before the surgery. Of the 12 patients that were anti-HCV positive, only one patient was aware of his results before the surgery. Of the 117 patients that were HbS Ag positive, 32 patients were aware of their results before their surgery. These numbers may be somehow unreliable, as these diseases can be sexually transmitted and the people who know they have the disease (especially HIV) may be reluctant to express that they have an infectious disease. This is important especially in conservative countries where people who have these diseases (especially HIV and HCV) might be excluded from the society. It is a surgeon's most important responsibility to respect the patient confidentiality and to reassure the patient that his or her results will be kept confidential. Other than that, we have had patients who were reluctant to inform us about their disease before their blood work was positive for infection, which was mainly because they thought their disease would result in cancelation of their surgery. Thus, the surgeon must understand the patients concerns and address any questions in their mind accordingly. Also, we had patients with low socioeconomic status who already were diagnosed in other places before we obtained blood tests from them. They were told that they had the disease in the place they were diagnosed, but failed to understand or did not pay attention to it, thus did not inform us about this. Thus, obtaining these blood works can be crucial in increasing patient awareness, especially in countries of low socioeconomic status. There may be many other reasons for the patients to be reluctant to tell a surgeon that they have to the disease. For these reasons we obtain these blood work on a routine basis from all our patients who we perform surgery.

For patients undergoing septoplasty under local anesthesia, it might also be useful to perform preoperative serological testing, as septoplasty is also associated with the risk of contamination. It was reported that Turkish healthcare workers in a hospital setting have a high risk of percutaneous injury/mucosal exposure, but overall awareness of such risk is low.^40^ Although the occupational risk to healthcare workers is lower in Turkey than in some other developing countries,^40^, ^41^, ^42^ studies show that there is a high incidence of the exposure to blood borne pathogens in Turkish HCWs, as compared to developed countries.^40^, ^42^, ^43^, ^44^, ^45^ Some studies indicate that routine serological testing is not necessary for all preoperative patients, but should be performed in patients with risk factors.^23^ This approach could be feasible in low prevalence countries, but not in Turkey, which has higher prevalences. Furthermore, overall awareness of percutaneous and mucocutaneous injury among Turkish healthcare workers is low.^40^ Educational programs for increasing awareness of the risk in Turkish hospitals is of utmost importance and when combined with serological testing, such programs could be more effective and further increase awareness. To the best of our knowledge the present study is the first to assess the prevalence of HBV, HCV, and HIV infection in patients undergoing septoplasty. Also, our study includes a large patient population.

## Conclusion

Septoplasty with and without turbinate surgery is associated with the risk of transmission of blood–borne virus transmission to surgeons, whether performed under local or general anesthesia. The present findings indicate that education of healthcare workers combined with routine preoperative serological testing in patients undergoing septoplasty under general and local anesthesia could further increase patient and healthcare worker awareness, and decrease contamination rates.

## Funding

The authors disclose no financial relationship relevant to this publication.

## Conflicts of interest

The authors declare no conflicts of interest.
